# *In vitro *antimalarial drug susceptibility in Thai border areas from 1998–2003

**DOI:** 10.1186/1475-2875-4-37

**Published:** 2005-08-02

**Authors:** Wanna Chaijaroenkul, Kesara Na Bangchang, Mathirut Mungthin, Stephen A Ward

**Affiliations:** 1 Faculty of Allied Health Sciences, Thammasat University, Rangsit, Patumthani 12121, Thailand; 2 Department of Parasitology, Phramongkutklao College of Medicine, Ratchathewi, Bangkok 10400, Thailand; 3 Division of Molecular and Biochemical Parasitology, Liverpool School of Tropical Medicine, Pembroke Place, Liverpool L35QA, UK

## Abstract

**Background:**

The Thai-Myanmar and Thai-Cambodia borders have been historically linked with the emergence and spread of *Plasmodium falciparum *parasites resistant to antimalarial drugs. Indeed, the areas are often described as harbouring multi-drug resistant parasites. These areas of Thailand have experienced significant changes in antimalarial drug exposure patterns over the past decade. This study describes the *in vitro *antimalarial susceptibility patterns of 95 laboratory-adapted *P. falciparum *isolates, collected between 1998 and 2003,.

**Methods:**

Ninety five *P. falciparum *isolates were collected from five sites in Thailand between 1998 and 2003. After laboratory adaptation to *in vitro *culture, the susceptibility of these parasites to a range of established antimalarial drugs (chloroquine [CQ], mefloquine [MQ], quinine [QN] and dihydroartemisinin [DHA]) was determined by the isotopic microtest.

**Results:**

Mefloquine (MQ) sensitivity remained poorest in areas previously described as MQ-resistant areas. Sensitivity to MQ of parasites from this area was significantly lower than those from areas reported to harbour moderate (*p *= 0.002) of low level MQ resistance (*p *= 000001). Importantly for all drugs tested, there was a considerable range in absolute parasite sensitivities. There was a weak, but statistically positive correlation between parasite sensitivity to CQ and sensitivity to both QN and MQ and a positive correlation between MQ and QN. In terms of geographical distribution, parasites from the Thai-Cambodia were tended to be less sensitive to all drugs tested compared to the Thai-Myanmar border. Parasite sensitivity to all drugs was stable over the 6-year collection period with the exception of QN.

**Conclusion:**

This study highlights the high degree of variability in parasite drug sensitivity in Thailand. There were geographical differences in the pattern of resistance which might reflect differences in drug usage in each area. In contrast to many other studies there were weak, but statistically significant positive, correlations between sensitivity to CQ and sensitivity to MQ and QN. Over the six years of sample collection, parasite sensitivity appears to have stabilized to these drugs in these sites.

## Background

Malaria is still a major health problem in Thailand, especially along the Thai-Myanmar and Thai-Cambodia borders where multi-drug resistant malaria is highly prevalent [1]. Chloroquine-resistant (CQR) parasites were first reported in the late 1950s from Southeast-Asia and South-America. Since those early reports CQR has spread throughout the malaria endemic countries of the world. The Malaria Control Programme of the Ministry of Public Health of Thailand was established in 1963. The objective of the control programme is to monitor the susceptibility of *Plasmodium falciparum *to currently used antimalarial drugs using both *in vitro *and *in vivo *tests with the ultimate goal of providing effective malaria control strategies and delaying the emergence of drug resistance [2]. Both approaches to the sensitivity assessment have their strengths and weaknesses [3]. The *in vitro *sensitivity monitoring system is considered a suitable system for assessing absolute sensitivity without the confounding influences of host-related factors, such as host immunity and drug pharmacokinetics. The results from *in vitro *tests, therefore, provide a more objective insight into inherent drug sensitivity than do *in vivo *tests. However, compared to the *in vivo *test, there are technical requirements which make this type of analysis operationally more difficult.

Several *in vitro *sensitivity test systems have been developed and applied to sensitivity monitoring of *P. falciparum *in endemic areas. These include traditional *in vitro *tests based on the measurement of the effect of drugs on the growth and development of malaria parasites, *i.e*., schizont maturation or growth inhibition [4,5], incorporation of radiolabeled precursors [6], enzymatic activity of parasite lactate dehydrogenase (pLDH) [7] or histidine-rich protein II (HRP II) [8]. The *in vitro *sensitivity test based on the standard micro-technique recommended by the World Health Organization [5] using the schizont maturation inhibition test has been applied successfully in certain highly multi-drug resistant areas of Thailand. However, the technique requires immediate laboratory work at the sites, where availability of qualified personnel is not readily available. One approach to solve this problem is to collect patient's blood samples and perform the test at the central laboratories where qualified personnel exist. This approach requires preservation of parasite samples at low temperature (-196°C) in a liquid nitrogen tank and adaptation of the parasite isolates to short-term culture prior to *in vitro *sensitivity testing. An additional advantage of this approach is that it allows multiple assessment of drug susceptibility, increasing confidence in the data collected, compared to the one-off drug sensitivity analysis afforded by the use of freshly collected isolates. The main purpose of this study was to explore the *in vitro *susceptibility of recently collected and adapted *P. falciparum *isolates to chloroquine, quinine, mefloquine and dihydroartemisinin in order to establish the degree of variability in parasite sensitivity, any geographical patterns and the stability of parasite sensitivity over a six-year collection period.

## Methods

### Parasite isolates

**Figure 1:**
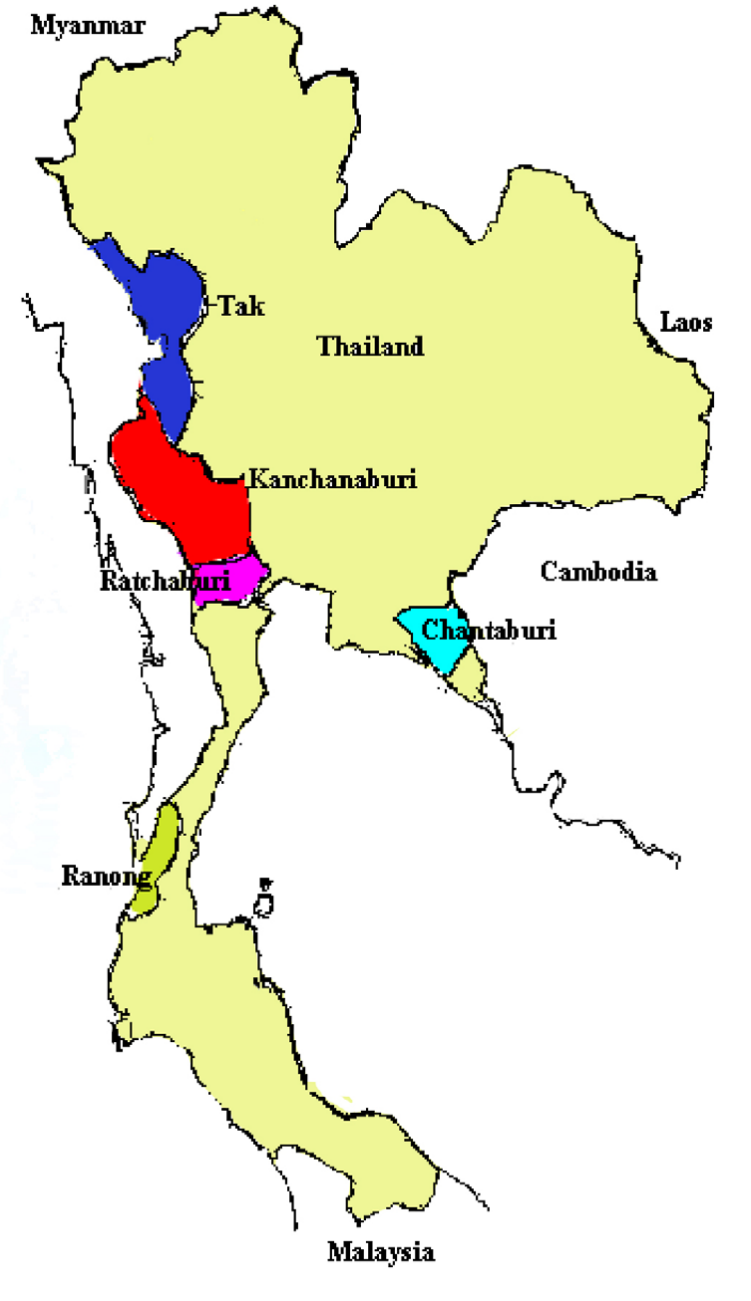
Map of Thailand showing the sampling sites: Tak, Kanchanaburi, Ratchaburi, Ranong and Chantaburi provinces.

This study was carried out between 1998–2003, *P. falciparum *isolates were collected from malaria endemic areas of Thailand (Figure 1), including the Thai-Myanmar border regions (Kanchanaburi, Tak, Ratchaburi and Ranong) and the Thai-Cambodia border (Chantaburi). Approval of the study protocol was obtained from the Ethics Committee of the Ministry of Public Health, Thailand. Fresh isolates of *P. falciparum *were collected from patients with acute uncomplicated falciparum malaria who presented at Mae Sot General Hospital and Malaria Clinics located in the various provinces. Inclusion criteria included those who had no previous history of antimalarial treatment within the preceding one month and with an asexual parasitaemia between 1,000 and 80,000/μl. All gave informed consent for study participation. Three to five millilitres of blood were collected into EDTA tubes from patients with a confirmed diagnosis of uncomplicated *P. falciparum *malaria. The fresh blood samples were then centrifuged to remove the buffy coat and cryopreserved in liquid nitrogen following the method of Rowe [9] before being transported to the laboratory.

Parasites were maintained in *in vitro *culture using the method of Trager and Jensen [10]. Briefly, infected blood samples were removed from liquid nitrogen tank storage and thawed in a 37°C water bath. An equal volume of sterile 3.5% sodium chloride was added, and the mixture was centrifuged at 1000 × g for 5 min. The pellet was washed three times and re-suspended with RPMI-1640 medium supplemented with 20% human serum and placed in a 25 ml tissue culture flask in a total volume of 10 ml containing a 5% RBC suspension. The flask was flushed with a gas mixture of 5%CO_2_, 5% O_2 _and 90% N_2 _and incubated at 37°C. The culture medium was changed once a day; group O red blood cells were added to maintain the 5% cell suspension. Parasite growth was monitored by Giemsa-stained (2% v/v, pH 6.8, 30 min) thin smear examination.

### *In vitro *sensitivity tests of *P. falciparum *isolates

Drug testing was performed using the cultured parasites once the parasitaemia of the culture reached an optimum density (0.5%–1% parasitaemia and 1.5% haematocrit). *In vitro *susceptibility was tested by monitoring [^3^H] hypoxanthine uptake [6]. Each drug was prepared as 10 mM stock solution and further diluted in RPMI-1640 medium to the desired concentrations. The plates (96-wells microtitre plates) were dosed with antimalarial drugs at a total of eight final concentrations as follows: mefloquine (1, 5, 10, 25, 50, 100, 150, 200 nM), chloroquine (5, 10, 25, 50, 100, 150, 250, 500 nM), quinine (10, 25, 50, 100, 150, 250, 500, 1,000 nM), and dihydroartemisinin (0.1, 0.2, 0.4, 0.8, 1.0, 5.0, 7.5, 10.0 nM). Ten microlitres of blood (1% parasitaemia, 20% haematocrit) was dispensed into each well of the sterile plates, followed by 100 μl of drug solution in RPMI-1640 without [^3^H] hypoxanthine. The plates were incubated at 37°C in a candle jar for 24 hours, pulsed with 5 μl of [^3^H] hypoxanthine solution (0.1 mCi/ml), and reincubated for an additional 24 hours. The plates were harvested using a Tomtec March III M semi-automatic harvester, using Wallac A Printed Filtermats (Finland), dried, and mixed with 5 ml of scintillation fluid. Each filter mat was then placed in a cassette and the radioactivity were measured using a 1450 MicroBeta Trilux liquid scintillation and luminescence counter (Wallac, Finland). The drug concentration that inhibited 50% parasite growth (IC_50_) were determined. Reported data represent the mean results from at least three independent drug sensitivity tests.

### Statistical analysis

The *in vitro *activity of antimalarials is presented as the geometric mean of the IC_50_s for all isolates. The Mann-Whitney U-test was used to determine whether the observed differences in the *in vitro *response of geographically distinct parasites to antimalarial drugs were significantly different. The potential for *in vitro *cross-resistance was evaluated by standard linear regression analysis. For all statistical tests the significance level (*p*) was set at 0.05.

## Results

### *In vitro *susceptibility of *P. falciparum *to antimalarials

**Figure 2:**
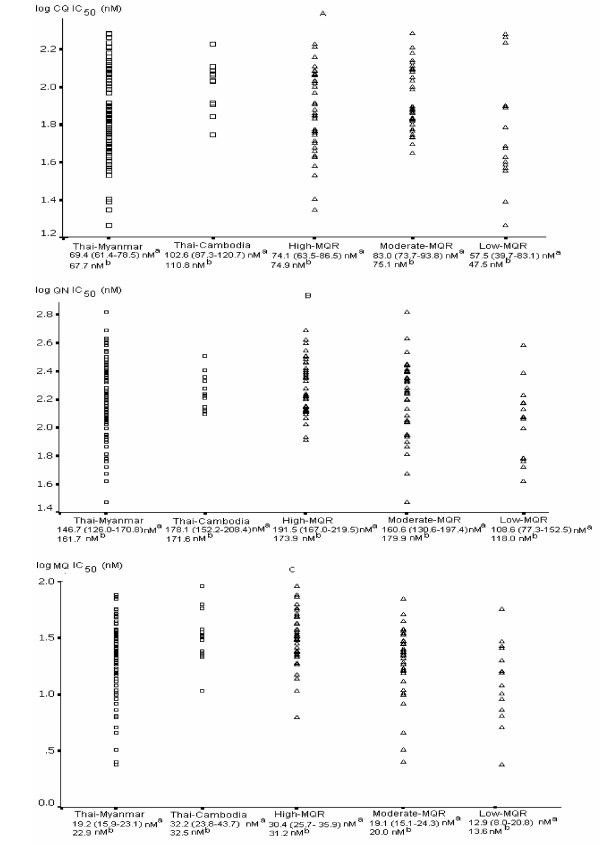
The scatter plot between the IC_50 _(nM) and the mefloquine resistance areas and the border regions. A represents the chloroquine data, B the quinine data and C the mefloquine data. (a) is the geometric mean 50% inhibitory concentration *in vitro *with the 95% CI and (b) is the median.

A total of 95 *P. falciparum *isolates were collected from five malaria endemic areas of Thailand: Mae-sot District, Tak (27), Ratchaburi (1), Kanchanaburi (37), Ranong (16) and Chantaburi Province (14). The *in vitro *susceptibility data are shown in Figure 2A to 2C. The geometric mean 50% inhibitory concentration (IC_50_) and 95%CI were 74.3 (45.2–122.2), 162.9 (91.9–288.8), 22.2 (10.8–45.6) and 1.35 (0.73–2.47) nM for CQ, QN, MQ and DHA respectively. There were no major differences in drug susceptibility over the six years the samples were collected with the exception of quinine sensitivity, which was lower in the isolates collected in 2003 (Table 1).

**Table 1:** *In vitro *susceptibility of Thai *P. falciparum *isolates according the years

Year	GMIC_50 _CQ (nM)		GMIC_50 _QN (nM)		GMIC_50 _MQ (nM)		GMIC_50 _DHA (nM)	
	Mean	95%CI	Mean	95%CI	Mean	95%CI	Mean	95%CI
1998	72.5	56.7–89.7	178.3	121.1–262.4	20.1	11.7–34.5	1.07	0.82–1.39
2000	65.1	44.8–94.5	243.4	163.7–361.8	28.1	15.8–50.1	1.64	0.92–2.92
2002	67.8	53.2–86.5	184.7	152.1–224.5	30.4	25.5–36.1	1.46	1.16–1.84
2003	79.1	68.8–90.1	136^b^	115.2–160.4	19.1	15.3–23.7	1.35	1.12–1.63

^a ^Geometric mean 50% inhibitory concentration *in vitro*

^b ^Significant difference with 2000 (*p *= 0.012) and 2002 (*p *= 0.024)

### *In vitro *susceptibility and geographical distribution

The malarious areas of Thailand have been categorized by the clinical level of mefloquine sensitivity *i.e*. high level MQ resistance (cure rate of MQ 750 mg is less than 50%; Tak and Chantaburi provinces), moderate level MQ resistance (cure rate between 50% and 70%; Kanchanaburi and Ratchaburi provinces) and low level MQ resistance (cure rate more than 70%; Ranong province). These groupings were maintained in the isolates studied here *in vitro*. Interestingly, parasites from the least MQ resistant area tended to demonstrate highest susceptibility to the other drugs tested.

### *In vitro *cross-resistance

**Figure 3:**
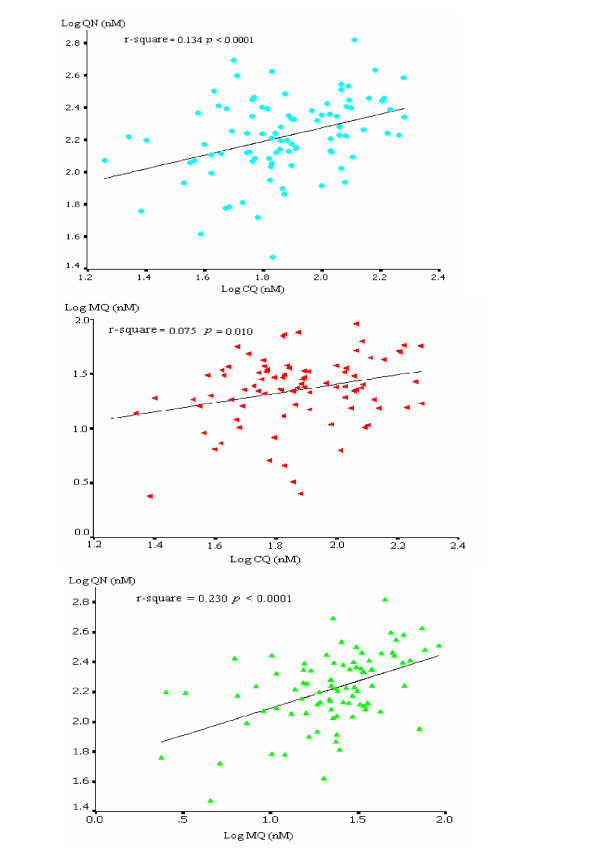
The scatter diagram and the regression line representing the relationship between IC_50 _(nM) values of CQ, QN and MQ.

In contrast to previous studies conducted on African and Thai isolates [11,12], there was a significant positive correlation between the *in vitro *susceptibility to CQ and susceptibility to MQ (*n *= 91, Pearson *r *= 0.075, *p *= 0.010) and QN (*n *= 93, Pearson *r *= 0.134, *p *< 0.0001) (Figure 3). Similarly there was a significant positive correlations between IC_50 _values of QN and the IC_50 _values of MQ (*n *= 91, Pearson *r *= 0.230, *p *< 0.0001). The strong correlation between the activities of MQ and QN was not surprising given the structural similarities between these two drugs and has been noticed by several authors [13,14]. In contrast, susceptibility to DHA did not correlate with susceptibility to any of the other drugs tested (data not shown).

## Discussion

In response to concerns about the malaria situation in Thailand, the government established the Malaria Control Programme since 1963. This programme has monitored and modified Thai policy on malaria control which was last revised in 1995. The major aim of the programme is to monitor the evolution of drug resistant malaria parasites [2]. The ability to implement a quick and relatively cheap assay to predict drug susceptibility of *P. falciparum *infections is vitally important, so that the spread of drug resistant parasites can be monitored and controlled. The first report of CQ resistance came from Thailand's borders in the late 1950s. More recently, MQ resistance was shown to develop rapidly after its introduction into clinical practice. This problem has been remedied by the introduction and deployment of the artemisinins in combination with MQ. Experience with CQ has highlighted the utility of drug sensitivity assays in plotting the emergence and spread of resistance. More recently molecular tools have found utility. For example the use of the K76T mutation in *pfcrt *gene as a marker for CQ susceptibility is the most useful molecular marker for CQ resistance, however, some parasite isolates that are sensitive to CQ also carried this mutation [11,15] and the use of this strategy is based on a full knowledge of all potential resistance mutations. Furthermore, the large variability in parasite sensitivity to drugs such as CQ suggests that although one molecular event may be the dominant control of susceptibility, other molecular events must contribute to this variability [16].

There have been many studies looking at parasite chemosensitivity in S.E.Asia. In general, the pattern of sensitivity shows consistency across the region, although parasite responses in parasites from Vietnam tend to show better responses to drugs [17,18]. Decreased susceptibility to quinine was first reported at the beginning of the 1980s in patients living near the Thai-Cambodia border [19,20]. The data reported in this current study indicate that the *P. falciparum *susceptibility to QN is stable or may have slightly improved recently. With respect to the other drugs studied against parasite sensitivity apperas to have stabilized.

Several studies have reported evidence of cross-resistance among certain group of quinoline-containing antimalarial drugs, *i.e.*, QN, MQ and HF, while showing an inverse relationship between this group of drugs and sensitivity to CQ [14,21-24]. Similarly, in other studies, the sensitivity of *P. falciparum *field isolates to the two structurally related drugs QN and MQ was strongly correlated [13,14,25]. This pattern of cross-resistance was also confirmed in this study. In contrast, a weak, but statistically significant, positive correlation was observed between sensitivity to CQ and sensitivity to either MQ or QN. The mechanisms behind this observation remains to be determined, but it does suggest that additional factors must be operational in these parasites compared to those displaying a clear negative correlation in their susceptibility patterns. Although a number of reports showed a positive correlation between *in vitro *susceptibilities of DHA and quinolines such as mefloquine and quinine, this cross-resistance was not apparent in this study [11,12,25]. In terms of distribution within Thailand, parasites from the Thai-Cambodia tended to be more resistant to all drugs evaluated compared to parasites from the Thai-Myanmar border.

The *in vitro *sensitivity data from adapted parasites showed the clearer view of drug profiles, which allows multiple assessment of drug testing. The drug profile in this study showed that the pattern of MQ resistance was spread to most of endemic areas, especially along the borders. The old drug policy was based on the level of MQ resistance areas, which treated the falciparum malaria with single dose of MQ 750 mg in low MQ resistance area, whereas the moderate and high MQ resistance areas were treated with multiple dose of MQ 750 mg plus 300 mg and 500 mg of artesunate at 6-hour interval, respectively [2]. From the fact of increasing the MQ resistance, the malaria control policy has been revised, which aim to reduce the MQ resistance. Thus, the combination of artemisinin derivatives with MQ has been introduced as first line drug for treating non-severe falciparum malaria throughout the country. However, QN still remains the first line drug for severe malaria treatment [26].

## Conclusion

This study indicates a relative stability in parasite susceptibility to four key drugs in the border areas of Thailand. Notably, resistance to CQ and MQ remains at a high level although there is substantial variation. There are indications that QN sensitivity may have increased slightly and, importantly for combination chemotherapy, parasite susceptibility to the artemisinin derivative, DHA remains high. This supports the decision of the Thai malaria control programme to introduce artemisinin containing MQ combinations as first line malaria treatment for the country.
